# A puzzle form of a non-verbal intelligence test gives significantly higher performance measures in children with severe intellectual disability

**DOI:** 10.1186/1471-2431-8-30

**Published:** 2008-08-01

**Authors:** Katrina D Bello, Nahal Goharpey, Sheila G Crewther, David P Crewther

**Affiliations:** 1 Brain Sciences Institute, Swinburne University, Hawthorn, 3122, Australia; 2 School of Psychological Science, La Trobe University, Victoria, 3086, Australia

## Abstract

**Background:**

Assessment of 'potential intellectual ability' of children with severe intellectual disability (ID) is limited, as current tests designed for normal children do not maintain their interest. Thus a *manual puzzle *version of the Raven's Coloured Progressive Matrices (RCPM) was devised to appeal to the attentional and sensory preferences and language limitations of children with ID. It was hypothesized that performance on the book and manual puzzle forms would not differ for typically developing children but that children with ID would perform better on the puzzle form.

**Methods:**

The first study assessed the validity of this puzzle form of the RCPM for 76 typically developing children in a test-retest crossover design, with a 3 week interval between tests. A second study tested performance and completion rate for the puzzle form compared to the book form in a sample of 164 children with ID.

**Results:**

In the first study, no significant difference was found between performance on the puzzle and book forms in typically developing children, irrespective of the order of completion. The second study demonstrated a significantly higher performance and completion rate for the puzzle form compared to the book form in the ID population.

**Conclusion:**

Similar performance on book and puzzle forms of the RCPM by typically developing children suggests that both forms measure the same construct. These findings suggest that the puzzle form does not require greater cognitive ability but demands sensory-motor attention and limits distraction in children with severe ID. Thus, we suggest the puzzle form of the RCPM is a more reliable measure of the non-verbal mentation of children with severe ID than the book form.

## Background

Intellectual disability (ID) affects 1.25% of the Australian population [1] and is defined according to the ICD-10 criteria as ongoing difficulties in age appropriate functioning and below age average cognitive performance as demonstrated by a score of two standard deviations below the mean on standardized intelligence tests. However, standardized intelligence tests such as the WISC-IV are often limited in their assessment of children with severe intellectual disability (ID) who are often unable to stay on task for the lengthy administration of the test, or handle its heavy reliance on language skills [2-4] and lack of ability to motivate [5]. Thus, to produce a valid measure of cognitive ability for children with severe ID, testing procedures must accommodate their profound deficits in communication, attention and social skills [6-10]. Such procedures are necessary and important to facilitate the most appropriate educational placement to enhance their educational and learning potential. We suggest that the Raven's Coloured Progressive Matrices Test (RCPM [11]) is a potentially more suitable alternative to tests like the WISC as it is an untimed non-verbal measure of reasoning ability [3,12,13]. This is supported by a recent study by Dawson, Soulières, Gernsbacher and Mottron [14], which showed that the WISC-III underestimates intelligence in children with ASD. They found that scores of 38 children with ASD were on average 30 percentile points higher on the Raven's Progressive Matrices (RPM) than their scores on the WISC-III, whereas no such difference was found for typically developing (TD) children. The RCPM consists of 36 coloured multiple choice matrices (although colour is irrelevant to the completion of the task), organized in three increasingly complex sets [3,11-15]. It is being utilized increasingly with children with severe ID, including those with Autism Spectrum Disorder (ASD) [5,16] in research settings to control for non-verbal mentation [13,17,18] and in educational settings to determine the level of functioning and treatment progress as part of a battery of tests [19,20].

Despite it being a better indicator of non verbal cognitive ability than the WISC III, many children with severe ID still show difficulties in completing the RCPM. Clark and Rutter [16] found that motivation and associated disruptive behaviours such as task avoidance, self-stimulation and escape behaviours in children with lower functioning ASD, hindered test performance on the RCPM. Techniques adopted to maintain motivation (e.g. lowering task difficulty to increase success rate in low scoring children) led to better performance, which suggests that the task itself is not sufficiently engaging of attention for children with impaired intellectual functioning. The standard book form of the RCPM also requires the child to point to their chosen pattern, which is a problem as pointing is one of several delayed social communication skills observed in many children with ID, particularly ASD [21].

To enhance compliance in cognitively less able clinical groups, Raven produced a board form of the RCPM [15] where each item, presented on a wooden board, can be completed with the correct placement of movable pieces. Raven et al. [15] claim that the board form is a consistent, reliable and psychologically valid estimate of reasoning ability, with a test retest reliability of approximately *r *= 0.80. However, although past studies [16,22-24] have utilized the board form, the study details are not available and, evidence of its validity is limited. Furthermore its heavy inflexible wooden design is often unsuitable for use for children with severe ID. Carlson and Weidl used a test-retest design to show that the board form produced better performance than the book form in typically developing children [22] and children with ID [23]. However, because they allowed for trial and error in the completion of the board form, it is unclear whether the better performance on the board form was due to increased opportunity for self-correction or the nature of the board form itself. The board form is also limited as the moveable pieces are easily disarranged when in use and administration of 36 separate board pieces is quite time consuming [15]. Such task characteristics do not encourage sustained attention and motivation in children with severe ID.

In line with the merits of the board form and considering its administrative inflexibility we have designed a puzzle version as an alternate form of the RCPM specifically designed to encourage greater sensory attention and motivation, increase task comprehension and consequently limit other disruptive behaviours in order to obtain a more valid measure of reasoning ability in children with ID. This new form resembles a jigsaw puzzle and therefore minimizes verbal task instructions for children with severe ID [25]. It is also conceptually like the board form in that participants must physically remove pieces, however, our puzzle form utilizes a cardboard and Velcro™ system to allow the children to simply grasp and easily remove their chosen piece and place it in the gap of the larger pattern. Unlike the board form, the puzzle form is presented in a folder with each item displayed individually on one page and each piece secured with Velcro to minimize weight, distractions and ease and time of administration. Another advantage of the puzzle form is that grasping the pieces maintains attention better than the requirement of pointing, as in the book form. This is consistent with the idea that grasping requires more brain activation than visual recognition alone [26]. Grasping requires processing of spatial location, in addition to form, orientation and size [27] and serves to draw attention to the object, which maintains attention on the task. Motor engagement with the pieces and placement in the appropriate area provides immediate feedback and requires more attentional resources. Kaplan et al. [28] showed that people with ID receiving sensory input from different pieces of equipment, showed less aggression and self-stimulatory behaviour and more task completion. This effect was also generalized to subsequent tasks, which supports the effect of tactile stimulation in increasing task engagement in people with ID. Motor engagement is particularly important in children with severe ID and children with ASD who are less motivated by social reinforcement [29] perhaps due to they failure to orient to and engage with the affective expressions of others [28,30,31]. Doussard-Roosevelt, Joe, Bazhenova and Porges [32] found that children with ASD were more engaged when their mothers physically and non-verbally demonstrated an object to them than when she verbally described the object to them.

Thus the aims of these studies were in Study 1, to test the validity of performance of typically developing (TD) on the puzzle form of the RCPM by comparing it to the standard book form; and in Study 2, to examine overall performance and completion rate of the puzzle and book form in children with idiopathic ID, Down Syndrome (DS) or ASD to establish the potential applicability of this alternative puzzle form to children with severe ID. We hypothesized that, in Study 1, TD children would show comparable performance in the book and puzzle form of the RCPM, irrespective of which form was completed first on a counterbalanced cross over design over a three week period. We also hypothesized that, in Study 2, children with severe ID, whether ID, DS or ASD, who completed the puzzle form, would show a higher performance rate than children who completed the book form, irrespective of clinical group.

## Study 1: Comparison of the standard and puzzle forms for the validation of the puzzle form of the RCPM

### Methods

#### Participants

Seventy-six typically developing (TD) children attending a mainstream primary school within the Catholic education system in the northeastern suburbs of Melbourne, Australia, participated in the current study. Participants were aged between 5 and 11 years (*M *= 8.57 years, *SD *= 2.06 years), 40 of whom were male, and 36 were female. Participants were required to speak English as a primary language and fall within the middle range for socio-economic status backgrounds. Participants had no known neurological intellectual disabilities and were screened for hearing problems and for normal or corrected to normal vision. Participants were randomly assigned to a group who complete the book form first or another group who completed the puzzle form first. Table 1 shows the chronological age and RCPM score of each group. As can be seen, the groups were closely matched and were not significantly different for age, *t*(74) = 0.45, *p *> .05.

**Table 1:** Means and standard deviations for age for children who were all randomly assigned to one of two groups that either completed the standard book form first or the puzzle form first.

**Group**	**N**	**Age (years) (SD)**	**RCPM score (SD)**
Total	76	8.6 (2.1)	25.6 (6.1)
Book form	38	8.7 (2.1)	25.5 (5.7)
Puzzle form	38	8.4 (2.1)	25.8 (6.7)

Ethics approval for studies 1 and 2 was obtained from the Swinburne University of Technology and La Trobe University Human Ethics Committees. Permission to conduct testing in the school was obtained from the Catholic Education Office in Victoria, and the Principal of the School. Individual parental or guardian consent for each child was required prior to testing and all children were free to withdraw from testing at any time.

#### Materials

The RCPM is comprised of 36 items divided into three subsets of 12 items (Sets A, Ab, and B). Each item consists of a different coloured pattern with six possible pieces available to fill the "missing" location required to complete the pattern. The participant's task was to deduce the theme of relations expressed among the designs and choose the missing figure from among the alternative set of six. The original book form displayed each item on a page in a booklet. The alternative puzzle version was the same size and colour as the book form, but differed in that each of the alternative patterns could be removed and physically attached to the missing place on the matrix through the use of a Velcro system.

#### Procedure

The standard administration procedure as prescribed by Raven et al [9], was used for the original book form, with trained clinicians administering both book and puzzle forms individually to each child [11,15], within the school setting. As suggested by Raven et al. no time limit was assigned for either task. Participants were required to select a piece from six alternatives that completed the pattern for each item by either pointing to their chosen response in the book form or by removing their chosen response and placing it in the missing section of the matrix in the puzzle form. Participants were asked to do this using the verbal instruction "find missing". This very simple, clear and short verbal instruction was chosen to ensure that it could be successfully used with children with ID who have limited receptive language. Participants were required to select a piece their responses by pointing to Item one of the standard and puzzle versions served as a practice trial, where incorrect responses were corrected and no further assistance or verbal reward was given during performance and completion of the task. Performance on the RCPM was calculated according to the number of items correct, and unattempted items were classified as incorrect. Inclusion criteria required children to attempt at least one full set of 12 items. Children attempting less than this, were excluded from further analyses.

In Study 1, the TD children were randomly assigned to two groups where one group attempted the book form first while the other half attempted the puzzle form. The alternate form of the RCPM was again administered after three weeks. To minimize the impact of maturation in learning and memory or practice effects on performance a three-week interval between the puzzle and book form was used [13,33].

#### Data Analyses

To validate the puzzle form, the performance of children who completed the standard book form first was compared to the performance of children who completed the puzzle form first using an independent samples t-test. A comparison of the two versions using a cross-over design was then used to examine the puzzle version performance over time, and to show that it matters little to overall performance of TD children, which form of the test was performed first. Previous test-retest studies using only the book form of the RCPM were conducted three weeks apart and reported correlations of Pearson's *r *= 0.80 [13,34,35]. As an alternative measure to Pearson's *r*, interclass correlation coefficient (*ICC*) [31] and coefficient of variation of measurement error (*CVME*) [31] were also calculated for an indication of degree of relatedness and percentage of variation respectively, between scores from the first and second test occasions.

## Results

Data were initially screened for outliers and any violations of the assumptions of normality, homogeneity of variance, and sphericity. No outliers or violations of assumptions in the data were detected.

### Comparison between performance on standard and puzzle forms

Table 1 shows the RCPM means and standard deviations for the TD participants who completed the original book form and the group who completed the puzzle version. It can be observed from Table 1 that the mean score for each group was similar and an independent samples t-test showed no significant difference in RCPM score between children who completed the original book form and children who completed the novel puzzle form, *t *(74) = -0.22, *p *> .05.

### Cross-over design

**Figure 1:**
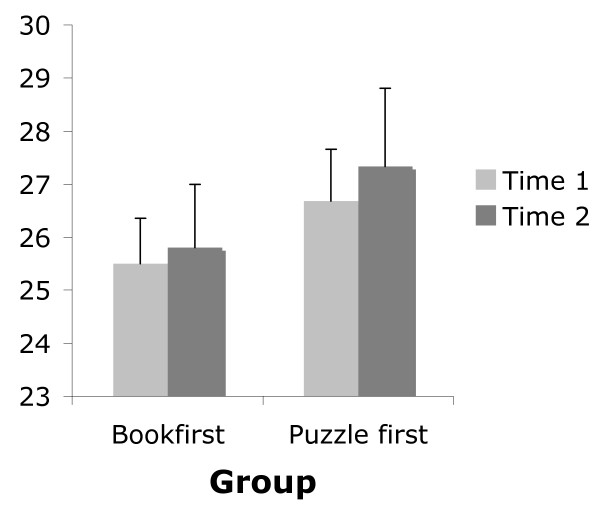
Mean and standard error of RCPM score for typically developing participants who completed the original book form first and those who completed the puzzle version first.

As displayed in Figure 1, the mean raw performance score for the first attempt was lower than for the second attempt irrespective of which version was completed first. A repeated measures ANOVA found this to be a significant effect, *F*(1, 74) = 8.62, *p *< .05. No significant interaction effect *F*(1, 74) = 0.14, *p *> .05 was found.

As presented in Table 2, a high correlation, *r *= 0.85, *p *< .01, was found between first and second attempt regardless of the form. The correlation between the first and second attempt for participants who completed the puzzle form first was higher, *r *= 0.93, *p *< .01, than for participants who completed the standard form first, *r *= 0.76, *p *< .01. This pattern was also observed with the ICC and CVME measures in that respectively, the degree of relatedness between first and second test occasions was greater for those who completed the puzzle form first compared to those who completed the book form first; and the percentage of variation between scores from the first and second test occasions was less in those who completed the puzzle form first compared to those who completed the standard form first.

**Table 2:** Correlation coefficients Pearson's r, interclass correlation coefficient (ICC), and coefficient of variation of measurement error (CV_ME_) values for RCPM score for first and second attempt for children who completed the book first and children who completed the puzzle first.

**Group**	**N**	**R**	**ICC**	**CV_ME_**
Total	76	0.85	0.82	7.22%
Book first	38	0.76	0.74	7.89%
Puzzle first	38	0.93	0.88	6.70%

A large test-retest reliability score (*r *= 0.85, *p *< .01) was found between the standard book form and the puzzle version, in TD children. This correlation is comparable to past studies solely examining the RCPM book form using a similar time frame of three weeks [32-34]. The findings suggest that the puzzle form is as useful as the standard book form of the RCPM in measuring nonverbal mentation in typically developing children.

In summary, the findings of Study 1 support the hypothesis that the book and the puzzle forms are measuring similar constructs in TD children. This suggests that the puzzle form, can be used with children with severe ID and potentially enhance performance and completion rate whilst still measuring the same constructs as the book form. Study 2 was conducted to examine the use of the puzzle form of the RCPM to measure non-verbal mentation in children with ID to evaluate the hypothesis that the puzzle form maintains attention in such children.

## Study 2: The puzzle form of the RCPM to measure non-verbal mentation in children with Intellectual Disability

### Method

One hundred and eighty-nine children with ASD, Down Syndrome (DS) or idiopathic ID, recruited from specialist schools in metropolitan Melbourne, Australia, were originally administered the book or puzzle form, but 25 participants were unable to complete a minimum of 12 items and were therefore excluded from further analyses. Table 3 shows the means and standard deviations for chronological age for the remaining 164 children, divided into clinical groups. Inclusion criteria from Study 1 were also used in Study 2. Participants were randomly assigned to be administered either the book form or puzzle form. The puzzle and book form were administered as detailed in Study 1.

**Table 3:** Means and standard deviations for age for each group of children with Autism Spectrum Disorder (ASD), Down Syndrome (DS), and idiopathic intellectual disability (ID).

**Group**	**N**	**Chronological Age (years) (SD)**
Total	164	10.7 (3.9)
ASD	101	9.7 (3.5)
DS	20	11.8 (3.7)
ID	43	10.6 (3.5)

Given that the data from this study were not normally distributed, non-parametric testing was used for all analyses.

### Results

**Figure 2:**
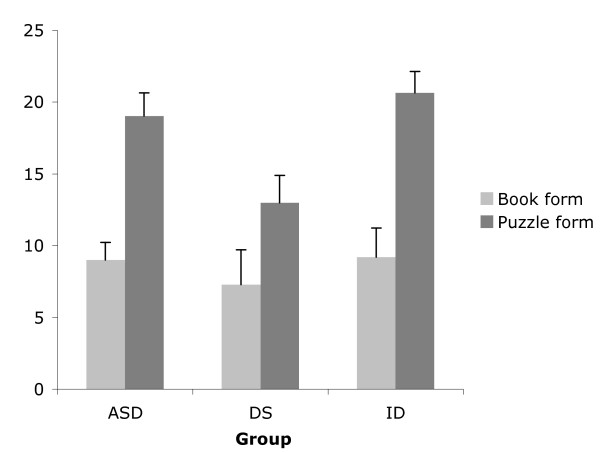
Mean RCPM score of children with ASD (n = 101), DS (n = 20), and ID (n = 43) who completed the book form or the puzzle form of the RCPM.

The RCPM means and standard errors for each ID group administered the book and puzzle forms are shown in Figure 2. A Kruskal-Wallis test showed no significant differences in RCPM score between the clinical groups, *H *(2) = 3.26, *p *> .05. A Mann-Whitney test showed a significant difference in RCPM score between performance on the book and puzzle form regardless of clinical group, *Z *= -5.54, *p *< .05. When each clinical group is examined separately, the ASD group participants who were administered the puzzle form performed significantly better than those who were administered the book form (*Z *= -3.99, *p *< .05) and the ID group (*Z *= -3.31, *p *< .05) but not the DS group (*Z *= -1.60, *p *> .05).

While the inter-group RCPM performance and hence mentation age was not significantly different, there was a significant difference in age between the three clinical groups *F *(2, 161) = 13.20, *p *< .05, with the mean age of the ASD group significantly less than the DS and ID groups. However, the age difference between children administered the puzzle and book form was not significantly different for each clinical group (ASD *t *(99) = -1.20, *p *> .05; DS *t *(18) = -0.78, *p *> .05; ID *t *(41) = 0.44, *p *> .05).

**Figure 3:**
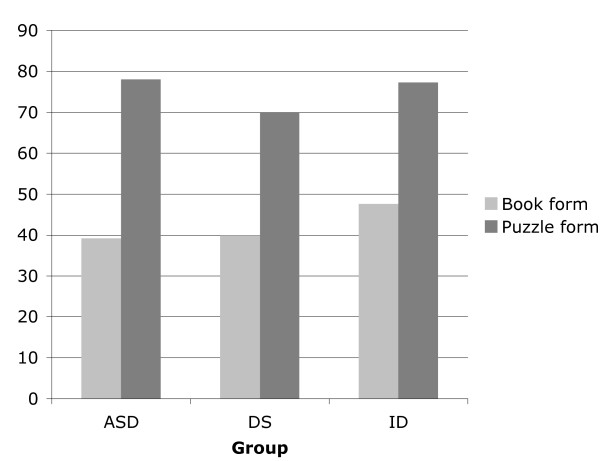
Percentage of children with ASD, DS, and ID who completed the book or the puzzle form of the RCPM.

As displayed in Figure 3, completion rate for the puzzle form (76.2%) was greater than for the book form (40%), regardless of clinical group. A Mann-Whitney test showed a significant difference in RCPM score between children who were able to complete the RCPM test and children who attempted at least 12 items but were unable to complete the task, regardless of which form they were administered, *Z *= -10.55, *p *< .05. Of those children who were unable to complete the book form, 55% of children with ASD, 68% of children with DS, and 67% of children with ID were able to complete the puzzle form. The results suggest that the use of the puzzle form as compared to the book from of the RCPM has resulted in better task performance and completion rate for all clinical groups.

**Figure 4:**
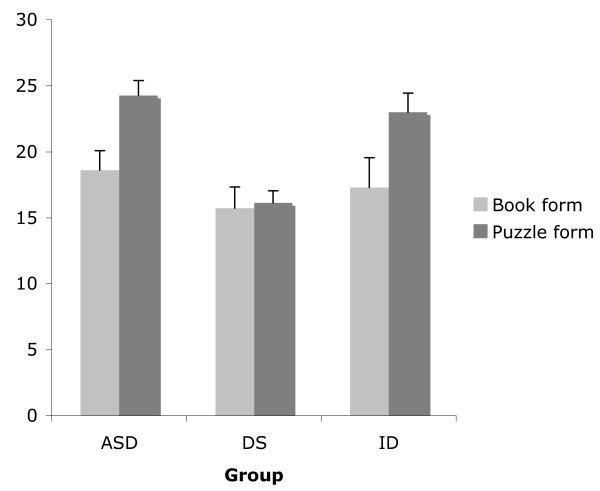
Mean RCPM score of children with ASD, DS, and ID who were able to complete the book form or the puzzle form.

To deal with the potential confound of completion rate, further analyses were performed only on participants who completed the puzzle or book form. A Mann-Whitney test showed that the participants who completed the puzzle form performed significantly better than those who completed the book form, *Z *= -2.89, *p *< .05. From Figure 4, it can be seen that in each clinical group, those who completed the puzzle form performed better than those who completed the book form, but only the ASD group showed this difference to be statistically significant (*Z *= -2.52, *p *< .05), but not the DS (*Z *= -0.19, *p *> .05) and ID (*Z *= -1.61, *p *< .05) groups.

## Discussion

The finding from Study 1 of no difference between the performance of TD children in the RCPM book and puzzle forms, combined with the finding of a strong correlation between first and second performance of the RCPM regardless of the order in which the forms were completed, shows that the alternative puzzle version is comparable to the book form in measuring reasoning ability. Past studies have reported that three factors delineate performance on the RCPM: continuous and discrete pattern completion, pattern completion through closure, and concrete abstract reasoning [23,36,37]. The high correlation between the book and puzzle forms found in the current study suggests that these constructs are maintained in the puzzle version.

Study 2 demonstrated that children with severe ID who were administered the puzzle form showed a performance advantage, as compared to those who were administered the book form. The findings suggest that the puzzle form provides a better indicator of learning potential than the book form in children with severe ID. We suggest that the performance advantage observed for the puzzle form is due to its unique features designed specifically to maintain attention and increase completion rate, though have not tested this suggestion directly. This is consistent with previous studies that have shown that added motivational techniques increased performance [5,18]. However, the current study does provide evidence that attention can be engaged while maintaining the underlying constructs being measured. Thus, it is likely that the puzzle form does not demand additional cognitive processing on children with severe ID, but increases sustained attention on the task in comparison to the book form. If this were the case, it would suggest that the puzzle form effectively engages cognitive ability of children with severe ID through the integration of motor and sensory based learning but only when the child directs their own responses. This is also advantageous as it potentially useful as it puts the emphasis on the test to be able to engage children with severe ID rather than requiring the administrator efforts to promote engagement in the child during the testing. For example, a study found that certain adult style of interaction, such as following a child's line of action instead of trying to re-direct it enhances social engagement in children with ASD [14].

The greater completion rate for the puzzle form than the book form for the children with severe ID in study 2 suggest that the ability to sustain attention and maintain motivation is a factor contributing to performance on cognitive ability measures for children with severe ID. This is supported by previous research showing that salience of variables engaging attention are highly correlated with measures of intelligence [38,39]. The findings challenge the clinical assumption that task incompletion reflects the inability to maintain attention and indicates limits of cognitive ability. Increasing task completion rate in children with severe ID is also important as it allows better comparison of cognitive ability to children with TD.

Alternatively, the performance advantage of the puzzle form may be due to the greater completion rate for children who were administered the puzzle form, compared to the book form. It can be argued that the puzzle form produces a performance advantage because the physical placement of response pieces reduces the mental function of abstractly visualizing the chosen piece in the missing area [23]. Unlike the results of the study by Carlson and Wiedl [23], a trial and error approach was not permitted and hence this cannot be the source of increased performance when using the puzzle form. In addition, the performance advantage in the puzzle form was only demonstrated by children of the same mental age, some with ID and some developing normally, which could suggest that the puzzle form maintained attention and motivation in those with severely limited attentional resources.

Given that more ID children were able to complete the puzzle form than the book form, it is possible that the performance advantage of the puzzle form was associated with an increased opportunity to select responses, as oppose to heightened task engagement. As the RCPM is a multiple choice task, the more items an individual completes, even at random, the greater the possibility of obtaining a higher overall scores. However, this is unlikely as additional analyses showed that the performance advantage of the puzzle form was maintained even when only those children who completed either RCPM form were included. However, this performance advantage was not observed in the DS and ID groups (also in the DS group when all participants were included regardless of whether they completed the RCPM or not). These non-significant findings are likely to reflect a Type II error and may be due to the small number of participants in the DS and ID groups. Future studies should examine more closely the effect of responses due to chance when completing the RCPM, specifically error-type analysis reflecting problem solving strategies in children with ID [40].

Profound deficits often make the assessment of children with severe ID very difficult, and the characteristics of standardized intelligence tests do not take into consideration such deficits. The current study indicates that children with ID perform better on the puzzle form of the RCPM and suggests that it is a better indicator of problem solving ability in children with severe ID than the book form. The puzzle form has proven to give a useful measure of RCPM in children with ID as it considers the degree of intellectual disability and severity of the language deficit, as well as engage attention and motivation while limiting distractions. Hence, this study supports the use of the puzzle form in clinical and educational research settings in place of the book form, as a better measure of reasoning ability in children with severe ID and in clinical settings for monitoring treatment progress, as a component of a battery of tests.

## Competing interests

The authors declare that they have no competing interests.

## Authors' contributions

KB and NG created the puzzle form of the RCPM and carried out the testing of participants and collation of data and manuscript drafting. SC designed the experiment and with DC has played a role in guiding the statistical analysis and manuscript improvement. All authors read and approved the final manuscript.

## Pre-publication history

The pre-publication history for this paper can be accessed here:


